# Antioxidant Potential of Pollen Polyphenols in Mitigating Environmental Stress in Honeybees (*Apis mellifera*)

**DOI:** 10.3390/antiox14091086

**Published:** 2025-09-05

**Authors:** Ivana Tlak Gajger, Aleksandar Cvetkovikj

**Affiliations:** 1 Department for Biology and Pathology of Fish and Bees, Faculty of Veterinary Medicine, University of Zagreb, 10000 Zagreb, Croatia; 2 National Veterinary and Food Institute, Faculty of Veterinary Medicine-Skopje, Ss. Cyril and Methodius University in Skopje, 1000 Skopje, North Macedonia; acvetkovic@fvm.ukim.edu.mk

**Keywords:** *Apis mellifera*, reactive oxygen species, honeybee detoxification mechanisms, bee bread composition, honeybee colony resilience, pesticide-induced stress, foraging behavior, thermal stress adaptation, honeybee nutritional requirements

## Abstract

Honeybee populations are increasingly threatened by various environmental stressors, including pesticides, pathogens, and climate change. Emerging research highlights the vital role of pollen polyphenols in supporting honeybee health through a network of antioxidants, immune responses, and detoxification mechanisms. This review synthesizes current findings on the chemical diversity, bioactivity, and functional relevance of polyphenolic compounds in honeybee nutrition. Pollen polyphenols, which include flavonoids and phenolic acids, possess remarkably high antioxidant potential, up to 235 times greater than that of nectar. They also significantly increase the expression of antioxidant enzymes, immune system genes, and detoxification pathways such as cytochrome P450s and glutathione-S-transferases. These compounds also demonstrate antimicrobial effects against key pathogens and mitigate the toxic effects of pesticides. The content and composition of polyphenols vary seasonally and geographically, impacting the resilience of honeybee colonies. Field and laboratory studies confirm that polyphenol-rich diets improve survival, gland development, and stress resistance. Advanced analytical techniques, including metabolomics, have expanded our understanding of polyphenol profiles and their effects on honeybee physiology. However, knowledge gaps remain in pharmacokinetics and structure–function relationships. Integrating this evidence into conservation strategies and good beekeeping practices, such as habitat diversification and targeted feed supplementation, is crucial for maintaining honeybee health and ecosystem services in a rapidly changing environment.

## 1. Introduction

Honeybees (*Apis mellifera*) face numerous environmental challenges that induce oxidative stress, a physiological condition characterized by an imbalance between the production of reactive oxygen species (ROS) and the antioxidant defense mechanisms. This imbalance represents a significant threat to individual honeybee health and, by extension, colony strength and survival [1,2]. Oxidative stress occurs when normal metabolic processes or external stressors generate excessive free radicals that overwhelm the organism’s antioxidant capacity, leading to cellular damage and functional impairment [3,4,5]. Antioxidants are critical protective compounds that neutralize free radicals, mitigating oxidative damage to cellular components, including proteins, lipids, and nucleic acids. For honeybees, dietary antioxidants derived primarily from pollen and nectar constitute an essential component of the defense mechanism against oxidative stress [6,7]. Among these dietary components, polyphenols as particularly flavonoids, represent a diverse group of plant secondary metabolites with demonstrated antioxidant properties that may significantly enhance honeybee resilience to environmental stressors [8,9,10]. The importance of polyphenols in honeybee diets extends beyond their antioxidant capabilities, so they also contribute to immune function, nutrient absorption, and overall honeybee colony vitality. Studies indicate that adequate consumption of high-quality pollen can significantly improve honeybee resistance to diseases, enhance immune responses, and mitigate the adverse effects of environmental stressors [11,12,13]. The global decline in honeybee populations, along with rising environmental pressures from pesticide exposure, inadequate natural food sources, and climate change, necessitates a comprehensive understanding of all factors that support honeybee health, including the role of dietary antioxidants [14]. Despite growing evidence supporting the beneficial effects of pollen-derived antioxidants, a systematic review integrating recent findings on their specific mechanisms of action, bioavailability, and practical applications in beekeeping is still lacking in the scientific literature.

So, this review aims to synthesize current knowledge regarding pollen polyphenols and their role in honeybee antioxidant defense mechanisms against environmental stressors. Specifically, it aims to examine the biochemical basis of oxidative stress in honeybees, characterize key antioxidant compounds in their diet, analyze how climate change affects antioxidant availability, evaluate the influence of dietary antioxidants on immunity and stress resilience, and identify critical knowledge gaps to guide future research. Through this detailed analysis, we aim to enhance understanding of how dietary antioxidants can improve honeybee health and survival in increasingly challenging environmental conditions. The specific objectives of this review are to define oxidative stress in the context of honeybee physiology and biochemistry, identifying its primary endogenous and exogenous sources (including environmental stressors, pathogens, and pesticides) and detailing its consequences for individual honeybee physiology, immune function, and overall colony health; to characterize the key antioxidant compounds in the honeybee diet, focusing on polyphenols (flavonoids and phenolic acids) and essential vitamins (Vitamin C, Vitamin E), detailing their occurrence in pollen and nectar, their metabolic fate, and their mechanisms of action within the honeybee; to analyze the impact of climate change variables (e.g., global warming, altered precipitation, and elevated CO_2_) on floral diversity, pollen abundance, and the nutritional quality of pollen, specifically concerning antioxidant composition, and evaluate the potential consequences for honeybee nutrition, immunity, and stress resistance; to elucidate the role of dietary antioxidants in modulating honeybee immunity, including the enhancement of immune responses, influence on resistance to pathogens and parasites, and potential synergistic interactions between different antioxidant compounds; to examine the contribution of dietary antioxidants to honeybee resilience against specific environmental stressors, particularly pesticide-induced oxidative stress and challenges arising from climate change and habitat loss, and their effects on honeybee longevity and colony survival; to identify significant knowledge gaps, conflicting findings, and promising future research directions of honeybee antioxidant biology, including the need for studies on bioavailability and metabolism, and the potential for developing antioxidant-rich dietary supplements for the apiculture sector; and to summarize the key findings of the review and discuss their practical implications for beekeeping practices, honeybee conservation strategies, and future research priorities.

During the writing process, a structured literature search protocol was implemented, which included clearly defined search strategies, specific inclusion and exclusion criteria, and methodologies for data extraction to ensure the relevance and quality of the studies reviewed.

## 2. Implications of Oxidative Stress in Honeybees

### 2.1. Biochemical Basis of Oxidative Stress in Honeybees

Oxidative stress represents a fundamental imbalance between pro-oxidant and antioxidant systems, leading to potential cellular damage. At the molecular level, this process involves excessive production of ROS, including superoxide anion (O_2_^−^), hydrogen peroxide (H_2_O_2_), and hydroxyl radical (OH), which overwhelm endogenous antioxidant defense mechanisms [3]. This biochemical disruption appears to be particularly problematic for bees due to their high metabolic rate and extensive flight activity [15]. The primary sources of ROS in honeybee physiology include normal cellular respiration, immune responses to pathogens, and exposure to environmental toxins [16]. Mitochondrial respiration generates superoxide anions as natural byproducts of energy metabolism [17]. In healthy bees, these radicals are efficiently neutralized by endogenous antioxidant enzymes, including superoxide dismutase (SOD), catalase (CAT), and glutathione peroxidase (GPx) [18]. However, when ROS production exceeds antioxidant capacity, cellular damage accumulates through lipid peroxidation, protein oxidation, and DNA damage. Recent studies have demonstrated that flight muscle mitochondria in worker bees show particularly high susceptibility to oxidative damage, which may contribute to age-related decline in foraging capacity [15]. In honeybees, as in other aerobic organisms, ROS are continuously generated as byproducts of normal cellular metabolism, particularly through mitochondrial electron transport during respiration [15,19]. As was already mentioned, the honeybee genome encodes several enzymes involved in antioxidant defense, including SOD, CAT, and GPx, which work together to neutralize ROS [20,21]. Additionally, non-enzymatic antioxidants such as glutathione (GSH), vitamin C, and vitamin E provide complementary protection against oxidative damage. When environmental stressors disturb this balance, oxidative stress can lead to lipid peroxidation, protein oxidation, and DNA damage, ultimately compromising cellular function and health [22].

Honeybees encounter numerous sources of oxidative stress throughout their lifecycle. Environmental factors are primary stressors, with pesticide exposure representing a particularly significant threat. The relationship between pesticide exposure and oxidative stress has received considerable attention in the recent literature [23]. Neonicotinoids and organophosphates have been shown to induce oxidative stress in honeybees through mechanisms involving increased ROS production, which in turn modulates the activity of endogenous antioxidant enzymes [24]. Studies have shown that sublethal pesticide exposure can persist in bee tissues for weeks, creating chronic oxidative stress conditions that may compromise long-term health and survival [25]. Environmental toxins, beyond pesticides, also contribute to oxidative burden [26]. Pathogen infections also represent a substantial source of oxidative stress. Viral infections, like deformed wing virus (DWV) and acute bee paralysis virus (ABPV), along with parasitic infestations by *Varroa destructor* and fungal infections by *Nosema ceranae*, trigger oxidative responses in honeybees [27,28]. Alaux et al. (2010) demonstrated that *N. ceranae* infection significantly increases honeybee susceptibility to pesticide toxicity through oxidative stress mechanisms, highlighting the detrimental synergistic effects of multiple stressors [29]. Additionally, exposure to thiamethoxam in food negatively impacts honeybee queen reproductive physiology [30,31]. Nutritional stress from monoculture landscapes and limited floral diversity may contribute to oxidative imbalance by limiting the intake of dietary antioxidants [32,33]. Honeybees perform energetically demanding tasks, such as foraging, and experience elevated metabolic rates that lead to increased ROS production [15]. Climate change factors, including higher temperatures and extreme weather events, further exacerbate oxidative stress through direct physiological effects, the emergence of secondary diseases (in honeybees and wild bees) [34], and indirect impacts on nutritional resource availability [14]. Furthermore, management practices like migratory beekeeping and exposure to radiofrequency electromagnetic fields (RF-EMFs) have been shown to increase markers of oxidative stress [35,36,37]. Here, we synthesize and critically evaluate the current available scientific literature concerning the role of dietary antioxidants, with a specific focus on pollen-derived polyphenols and essential antioxidant vitamins, in honeybee health, particularly in the context of mitigating oxidative stress induced by environmental factors. So, the scope encompasses the biochemical underpinnings of oxidative stress in honeybees, the identification and function of key dietary antioxidants, the influence of environmental changes, and the intricate links between dietary antioxidants, immunity, and stress tolerance (Table 1).

### 2.2. Environmental Stressors Affecting Honeybees

Honeybees are affected by various environmental stressors that threaten their health and survival, including pathogens, pesticides, habitat loss, and climate change. They are vulnerable to bacterial pathogens causing diseases like American foulbrood, European foulbrood, spiroplasmosis, and septicemia. These pathogens have long infections and propagate rapidly, causing substantial harm that adversely affects honeybee growth and development [44]. Pesticides in agriculture aim to reduce pest and weed damage, but they can negatively impact honeybees by affecting their behavior, impairing antioxidant abilities, and reducing immunocompetence. Moreover, pesticides can increase the sensitivity of honeybees to viral infections, which compounds the risk of disease [45,46]. Different types of pesticides elicit varied responses from honeybees, with some exhibiting severe negative impacts on their health and behavior [44,47]. Climate change threatens honeybee populations by disrupting weather patterns and the availability of flowering plants. These shifts can cause nutritional stress due to misaligned foraging and food sources. Warmer fall and winter temperatures also extend foraging periods, complicating energy management for honeybee colonies during wintering. Recent reports indicate that managed honeybee colony losses have approached 40%, underscoring the severity of this issue [48]. The degradation of natural habitats further exacerbates the pressures on honeybees. Loss of foraging areas and nesting sites for wild bees limits their access to essential resources needed for survival and reproduction. This environmental change negatively impacts foraging efficiency, immune function, and overall colony health [46,47]. The interaction between these various stressors creates compounded effects on honeybee health. For instance, the presence of pesticides may increase honeybees’ vulnerability to diseases caused by pathogens, leading to a cyclical deterioration of their health [45,49]. Understanding the interactions among various stressors is essential for creating strategies to protect honeybee populations in changing environments. Recent decades have seen significant colony losses linked to these multifactorial stresses, which weaken hives and compound health issues. Studies show that colony failure often results from multiple factors, with oxidative stress playing a key mediating role [50].

### 2.3. Consequences on Physiology, Immunity, and Honeybee Colony Health

Oxidative stress has diverse effects on honeybee physiology, with particularly pronounced impacts on the nervous system. Evidence indicates that oxidative damage to brain tissues impairs cognitive functions essential for foraging, navigation, and learning, thereby compromising individual foraging efficiency and, consequently, honeybee colony nutrition [51]. Chronic oxidative stress has wide-ranging deleterious effects on honeybee physiology. At the cellular level, accumulated oxidative damage leads to inflammation, impaired energy metabolism, and accelerated senescence of tissues [21]. For individual bees, this can translate into shortened lifespan, reduced flight capability, and cognitive impairments (e.g., memory and learning deficits) due to neural damage. Oxidative stress also compromises the bee’s immune system; excessive ROS can dysregulate immune signaling or directly damage immune cells and enzymes, resulting in immunosuppression [21,50]. For example, habitat-related stress was shown to induce oxidative stress in bees alongside suppressed immune gene expression [52]. Consequently, oxidatively stressed bees become more susceptible to infections and parasites, creating a vicious cycle of declining health. Neurological function appears particularly vulnerable to oxidative damage, with implications for navigation, communication, and learning behaviors essential for foraging success. Studies have documented decreased performance in learning tasks among bees experiencing chronic oxidative stress, suggesting that cognitive impairment may contribute to reduced foraging efficiency and colony productivity [53]. In winter bees, whose extended lifespan is crucial for colony overwintering success, oxidative stress accelerates senescence processes, potentially reducing survival rates [54]. Immunologically, oxidative stress compromises honeybee defense mechanisms through several pathways. ROS overproduction impairs hemocyte function and antimicrobial peptide production, key components of the innate immune response [55,56]. Di Pasquale et al. (2013) showed that pollen quality affects tolerance to *Nosema* and that survival correlates with the activity of the immune enzyme phenol oxidase [6]. At the colony level, these individual effects result in reduced brood production, decreased honey yields, and ultimately, increased colony mortality [57]. Colonies experiencing oxidative stress exhibit altered division of labor, with premature transitions from hive to foraging duties disrupting the optimal age demographics of honeybee colony members [58]. Additionally, environmental stressors such as electromagnetic fields have been linked to behavioral alterations in colonies, suggesting that stress-induced plasticity may extend to changes in queen-related dynamics [59]. Oxidative stress reduces the resilience and productivity of both individual worker bees and the entire colony (Table 2).

## 3. Antioxidants in Honeybee Diet—Key Compounds and Their Sources

Bee pollen represents a concentrated source of diverse polyphenolic compounds that vary considerably based on botanical and geographical origin [66,67,68]. The total phenolic content in bee pollen can range dramatically, with some Brazilian samples reported as containing 1.7–2.2% [69], and other geographical regions reporting lower values, such as 720 mg/100 g (0.72%) [66]. This variation reflects the diverse floral sources from which bees collect pollen and the influence of environmental factors on plant secondary metabolite production [70,71]. The predominant polyphenolic compounds in pollen include flavonoids such as quercetin, kaempferol, isorhamnetin, rutin, and luteolin [66,67,72]. Quercetin levels can vary enormously between samples, ranging from 61.2 to 1221.7 mg/100 g, while (+)—catechin concentrations span from 66.75 to 337.39 mg/100 g [66]. Additional important compounds include phenolic acids like caffeic acid, p-coumaric acid, and chlorogenic acid, as well as specialized metabolites such as hydroxycinnamic acid amide derivatives and spermidine compounds [69,73,74]. The antioxidant activity of pollen correlates strongly with its polyphenolic content, particularly the presence of flavonols with specific structural features [70,72,75]. The planar structure of flavonols caused by hydroxyl groups at position 3 promotes higher radical capture through electron delocalization, while the number of hydroxyl groups directly influences their potency as electron scavengers [70,72]. Research has shown that each type of pure floral pollen has a consistent antioxidant capacity, regardless of its geographic origin. Approximately 50% of this activity is attributed to flavonoids and phenolic acids [76]. Modern extraction techniques such as microwave-assisted extraction can increase polyphenolic yields by 40–60% compared to conventional methods, enhancing the recovery of these bioactive compounds [77]. Pollen polyphenols play a crucial role in the antioxidant capacity, contributing significantly to its potential health benefits and its ability to mitigate environmental stress in honeybees. Both monofloral and polyfloral pollen samples are recognized as rich sources of polyphenols, with substantial antioxidant potential attributed to their complex composition [65,70]. These compounds are essential for mitigating oxidative stress, which is a critical factor in the health and survival of honeybees, particularly under environmental stressors. Pollen and nectar are primary sources of dietary antioxidants for honeybees, with polyphenolic compounds being a dominant class of bioactive constituents [78]. Comprehensive phytochemical analyses reveal substantial diversity in polyphenolic profiles across plant species, with pollen typically containing higher concentrations than nectar [76]. Pollen from families such as Rosaceae, Fabaceae, and Asteraceae has been associated with high antioxidant activity and phenolic content [79,80]. Flavonoids are the most abundant class of polyphenols in honeybee-collected pollen, with quercetin, kaempferol, and rutin consistently found across diverse botanical sources [81]. The predominant group of polyphenols found in bee pollen is flavonoids, which typically constitute about 0.25% to 1.4% of its total composition [21]. Quantitative analyses indicate that the total flavonoid content in honeybee pollen ranges from 2% to 5% dry weight, with significant seasonal and geographical variations reflecting local floral diversity [82]. Notably, the overall polyphenolic content in bee pollen ranges from 3% to 5%, with variations influenced by the origin of the raw material [73]. This variability is important because the antioxidant capacity of pollen samples often correlates positively with their polyphenol concentrations [83,84]. Multifloral pollen typically exhibits higher antioxidant potential compared to monofloral sources, highlighting the nutritional benefits of diverse foraging habitats for honeybee colonies [6]. Anthocyanins, which significantly contribute to the characteristic coloration of many pollen types, exhibit substantial free radical scavenging capacity in other plant products [85] and thus may confer oxidative protection to honeybees consuming diverse pollen diets.

### 3.1. Role in Honeybee Metabolism

Dietary antioxidants function as essential cofactors in honeybee metabolism, supporting both energy production and cellular protection [86]. The most significant antioxidant compounds in honeybee diets include ascorbic acid (vitamin C), tocopherols (vitamin E), carotenoids, and various polyphenolic compounds [44]. Each class of antioxidants contributes distinct protective mechanisms, and their synergistic interactions appear critical for optimal antioxidant protection [45]. Polyphenolic compounds represent the largest and most diverse group of dietary antioxidants available to honeybees. These compounds are concentrated primarily in pollen, though nectar and propolis also provide significant contributions. The polyphenolic profile of honeybee diets varies substantially based on floral sources, seasonal availability, and geographic location [45]. Following ingestion, pollen polyphenols undergo metabolic processing in the honeybee digestive system, with evidence suggesting selective absorption and biotransformation of specific compounds [8]. Pharmacokinetic studies indicate that p-coumaric acid, a phenolic acid abundant in pollen, is efficiently absorbed and distributed throughout honeybee tissues, including the brain, where it may provide neuroprotective effects against oxidative damage [8,87]. This biochemical support is crucial given that honeybees have a high metabolic rate and correspondingly high baseline ROS production during flight and thermogenesis [88]. Metabolically, dietary polyphenols influence critical detoxification pathways in honeybees. Experimental evidence demonstrates that p-coumaric acid upregulates cytochrome P450 enzyme expression [8], a key component of xenobiotic metabolism, which is discussed further in Section 6.1. Additionally, flavonoids appear to modulate energy metabolism in honeybees, with quercetin supplementation influencing mitochondrial function and ATP production in flight muscles [8]. Emerging evidence suggests that pollen polyphenols can significantly influence gut microbiome composition in honeybees. Previously published articles demonstrated that dietary quercetin and effective microorganisms (EM-1) alter core gut bacterial communities, potentially enhancing colonization resistance against opportunistic pathogens [89,90]. This microbiome-mediated effect represents an additional mechanism through which dietary polyphenols may influence honeybee health.

### 3.2. Mechanisms of Antioxidative Action

Pollen polyphenols exhibit powerful antioxidant activity through several well-characterized mechanisms that collectively protect against oxidative stress and cellular damage [44,91]. The primary mechanism involves direct free radical scavenging, where polyphenolic compounds neutralize ROS by donating hydrogen atoms or electrons to unstable molecules [92,93]. This electron donation stabilizes free radicals and prevents them from causing further oxidative damage to cellular components. The structural features of pollen polyphenols are crucial for their antioxidant effectiveness. Flavonoids with planar structures caused by hydroxyl groups at position 3 promote higher radical capture through enhanced electron delocalization, while the total number of hydroxyl groups directly influences their potency as electron scavengers [72]. The catechol group on the B ring, the 2,3-double bond, and conjugated groups on the C ring are particularly important structural elements that enhance antioxidant capacity [93,94]. Beyond direct radical scavenging, pollen polyphenols employ additional protective mechanisms, including metal ion chelation, where they bind and inactivate metal catalysts that promote oxidation reactions [44,95]. These compounds can also upregulate endogenous antioxidant enzymes and assist in repairing oxidative damage to biomolecules [72]. Research demonstrates that pollen extracts effectively protect cells against oxidative damage triggered by chemical free radical generators, with this protective effect directly attributed to the phenolic compounds’ ability to scavenge reactive species and prevent cellular injury [96]. The synergistic action of multiple antioxidant compounds in pollen, including flavonoids, phenolic acids, carotenoids, and vitamins C and E, creates a comprehensive defense system against oxidative stress [97,98]. This multi-component approach provides robust protection by targeting different types of free radicals and reactive species through complementary mechanisms, making pollen a potent natural antioxidant source [99,100]. Pollen polyphenols enhance oxidative stress resistance in honeybees through their antioxidant effects. They primarily work by scavenging ROS, which can cause cellular damage when excessive [49,83]. The excessive production of ROS can lead to oxidative stress, which is associated with various chronic diseases and accelerates the aging process [39]. In honeybees, factors such as environmental pollution, monoculture diets, and exposure to pesticides can increase ROS levels, thus compromising their immune defense [11,44]. One of the primary mechanisms is direct free radical scavenging, wherein the hydroxyl groups characteristic of flavonoid structures, such as those in quercetin, donate hydrogen atoms to neutralize ROS [101]. Structure–activity relationship studies demonstrate that flavonols with specific hydroxylation patterns, including quercetin, exhibit particularly potent radical scavenging capacity [102], which can be relevant to honeybee oxidative defense. Beyond these direct antioxidant effects, polyphenols also modulate endogenous antioxidant systems in honeybees. Dietary quercetin can mitigate imidacloprid’s impacts by lowering the pesticide burden in bees and by engaging detoxification pathways, while imidacloprid alone can alter antioxidant enzymes [103,104]. Similarly, Bryś et al. (2025) observed that pollen supplementation enhanced both SOD activity and glutathione levels in worker bees, establishing a link between dietary polyphenols and endogenous antioxidant capacity [21].

Research demonstrates that pollen consumption significantly enhances honeybee antioxidant enzyme systems, with the magnitude of benefit directly related to the phenolic and flavonoid content of the pollen source [21]. Studies show that bees fed pollen from plants rich in phenolic compounds and flavonoids, such as rapeseed, phacelia, buckwheat, and goldenrod, exhibit increased activities of SOD, CAT, GST, and GPx in both their fat body tissues and hemolymph compared to control groups [21]. The antioxidant capacity of pollen depends on various bioactive compounds, including phenolic acids, flavonoids, and specialized enzymes [74], which work synergistically to modulate antioxidant enzyme expression and activity in bee tissues. Specifically, enzymes like CAT and SOD, along with GSH, play crucial roles in the antioxidant effects observed when bees consume phenolic-rich pollen [74]. Experimental evidence shows that honeybees fed on different pollen types exhibit varying antioxidant responses, with some pollen sources producing higher antioxidant enzyme activity while others increase total antioxidant capacity [40]. The total antioxidant capacity appears closely related to the number and concentration of antioxidant compounds present in the pollen, suggesting that diverse antioxidant profiles provide comprehensive protection [40]. Metal chelation is another significant mechanism through which pollen polyphenols protect honeybees against oxidative damage. Flavonoids containing catechol moieties effectively chelate transition metals, such as iron and copper, preventing these metals from participating in Fenton reactions that produce highly reactive hydroxyl radicals [105]. This metal-chelating property may be particularly relevant in contexts where honeybees encounter environmental pollutants containing heavy metals. Additionally, certain pollen polyphenols directly influence gene expression through interaction with transcription factors, notably Nuclear factor erythroid 2-related factor 2 (Nrf2), which regulates antioxidant response elements in the genome [106]. This epigenetic mechanism may lead to long-term adaptations to oxidative stress by enhancing the activity of endogenous antioxidant pathways. Beyond enzyme enhancement, pollen consumption provides protective effects against oxidative damage in honeybee systems. The high content of flavonoids, particularly flavonols and anthocyanins, promotes healthy cellular function by lowering reactive oxygen species levels and mitigating their adverse effects on bee physiology [107,108]. This protective mechanism is particularly important for maintaining reproductive health and cellular integrity under environmental stress conditions.

### 3.3. Vitamins with Antioxidant Properties

Honeybees acquire essential vitamins (e.g., vitamin C and vitamin E) with antioxidant properties primarily through pollen consumption, with vitamin content varying substantially across botanical sources. Vitamin E is a vital lipophilic antioxidant, present in both pollen and bee bread [73]. The complex, comprising tocopherols and tocotrienols, provides critical lipid-soluble antioxidant protection in bee body cells [17]. These compounds are particularly important for protecting cell membranes and lipid-rich tissues from peroxidative damage [15]. Vitamin C (ascorbic acid) is another essential antioxidant in the honeybee diet, acting as an electron donor that neutralizes reactive oxygen species and regenerates oxidized vitamin E [109]. The concentration of ascorbic acid declines during storage due to oxidative degradation [76]. B vitamins, including riboflavin (B2), pyridoxine (B6), and pantothenic acid (B5), have antioxidant properties through their roles as enzyme cofactors in redox metabolism. These compounds support metabolic functions essential for oxidative homeostasis in honeybees and are found at nutritionally significant levels in diverse pollen types [110].

### 3.4. Dietary Sources and Their Bioavailability

The bioavailability of pollen antioxidants to honeybees depends on how effectively they can ingest and assimilate these compounds. Fresh pollen has a tough outer coat (sporopollenin) that can limit digestibility, but adult bees have evolved strategies to access pollen contents, mixing pollen with nectar and enzymes in the mouthparts, packing it into granules, and fermenting it into bee bread in the comb cells. Bee bread, which is fermented pollen, undergoes lactic acid fermentation. It was previously believed that this process significantly enhances nutrient availability. However, recent studies show that the nutritional value and antioxidant effectiveness of pollen do not significantly change during short-term storage. Bees benefit equally from both fresh pollen and bee bread in terms of their physiological effects [21]. The fermentation process primarily preserves pollen and may slightly increase certain vitamin levels (e.g., B vitamins), but polyphenols remain relatively stable and bio-accessible. Key antioxidants in pollen, such as flavonoids, are typically glycosylated (bound to sugars) in plants. When ingested, enzymes from the bee’s gut or its microbiota can hydrolyze these glycosides, releasing the active aglycones, which are more easily absorbed. Consequently, honeybee gut microbes may play a significant role in metabolizing dietary polyphenols, affecting their bioavailability and subsequent effects [111]. It is important to note that pollen diversity is significant for antioxidant intake, as different floral pollens contain unique profiles of polyphenols and vitamins [21]. A varied diet ensures a broad spectrum of antioxidants, potentially covering gaps where one plant’s pollen is deficient. Monofloral diets, especially from pollen with lower phenolic content (e.g., some wind-pollinated plants like pine), may provide fewer antioxidants than polyfloral diets [21]. Nevertheless, even monofloral pollen rich in polyphenols (e.g., buckwheat or rapeseed) can significantly boost bees’ antioxidant enzyme levels. The bioavailability of antioxidant vitamins in honeybees’ food depends on several factors, including pollen processing, storage conditions, and interaction with other dietary components. Experimental evidence indicates that bee bread preparation enhances vitamin bioavailability through microbial fermentation processes that partially degrade pollen walls and release encapsulated nutrients [86]. This predigestion process improves extraction efficiency during subsequent consumption by nurse honeybees, potentially maximizing antioxidant vitamin absorption. Seasonal and geographical factors significantly influence the availability of vitamins in honeybee diets. The nutritional composition of honeybee pollen has seasonal variations [112]. Similarly, agricultural intensification and resulting monocultures may limit access to specific vitamin-rich pollen sources, creating nutritional bottlenecks that compromise antioxidant defense [33,78]. Vitamin stability during storage represents another critical factor influencing bioavailability. Ascorbic acid content decreases significantly during pollen storage, with losses of up to 50% reported after three months [76]. Conversely, tocopherols demonstrate greater stability during storage, maintaining antioxidant potential over extended periods. These differential degradation rates suggest potential seasonal variations in vitamin availability for honeybee colonies.

### 3.5. Role in Honeybee Immune Support and Detoxification

Honeybee immunity is closely tied to nutritional quality, and antioxidants are now recognized as part of bees’ immune arsenal. High-quality pollen diets provide not only protein for immune enzyme and peptide synthesis but also antioxidants that shield immune cells from oxidative damage and even directly inhibit pathogens. The antioxidant system itself is considered one of the honeybee’s immune mechanisms [21], as it helps maintain immune homeostasis during infection (when ROS are produced as a defense but can also harm host tissues). Polyphenols like p-coumaric acid have been shown to activate the expression of antimicrobial peptide genes in bees, thereby enhancing disease resistance while simultaneously reducing oxidative stress [42]. This dual action improves the overall immune competence of the bee. Additionally, many plant polyphenols have inherent antimicrobial and antiviral properties. In vivo, feeding experiments demonstrate that pollen or supplements high in these compounds reduce pathogen loads: e.g., curcumin (a dietary phenolic from turmeric) significantly lowered *Nosema* spp. spore counts in infected bees and improved their survival [113]. Likewise, grape polyphenols (anthocyanins from pomace) fed to bees enhanced the expression of Relish, a key immune gene in the Toll pathway, and helped bees suppress deformed wing virus replication [114]. In terms of detoxification, antioxidants in the diet mitigate the harmful effects of xenobiotics. They can directly neutralize reactive metabolites of pesticides and prevent oxidative damage to the detoxification organs. More importantly, certain phytochemicals induce the transcription of detoxification enzymes (e.g., glutathione-S-transferases and P450s), which increases the bee’s capacity to metabolize and excrete toxins [42]. This has been demonstrated by studies of the same authors showing that bees consuming quercetin or p-coumaric acid along with pesticides had higher survival and lower toxicity symptoms than bees lacking those dietary phytochemicals.

## 4. Impact of Climate Change on Pollen Antioxidants Availability

### 4.1. Impact of Global Warming on Floral Diversity and Pollen Abundance

Climate change impacts plant and insect pollinator systems, significantly affecting pollen availability and its nutritional quality. Rising temperatures have shifted flowering phenology in many regions, with earlier bloom times disrupting the synchronization between bee foraging cycles and peak pollen availability [115]. These temporal mismatches may force bees to rely on suboptimal pollen sources or face periods of nutritional scarcity. Geographic range shifts of plant species create additional challenges for honeybee nutrition. As temperature zones migrate northward, traditional pollen sources may disappear from established bee territories while new plant species colonize these areas [116]. The antioxidant profiles of replacement plant species may differ substantially from historical sources, requiring bees to adapt to novel nutritional conditions without evolutionary preparation [14,29,117]. Extreme weather events, increasingly common under climate change scenarios, can dramatically reduce pollen availability during critical periods. Droughts, floods, and unseasonable temperature fluctuations can destroy flowering plants or interrupt their reproductive cycles [118]. These disruptions may create nutritional bottlenecks where bees face simultaneous increases in environmental stress and reductions in antioxidant availability [119]. An observed consequence is nutritional dearth for bees: when key forage plants produce less pollen (or flower at times when bee activity is low), colonies face periods of protein and micronutrient shortage [111]. Beekeepers worldwide have noted colony declines in unusually warm, dry years, often needing to supplement colonies with artificial feed due to lack of natural forage [111]. This reduction in pollen availability inherently means fewer dietary antioxidants for bees, since pollen is their main source of polyphenols and vitamins. Atmospheric carbon dioxide concentrations, a primary driver of climate change, appear to influence pollen production and composition directly [120]. Elevated CO_2_ levels generally increase plant biomass production but may dilute the concentration of protein and secondary metabolites in plant tissues [121]. This dilution effect could reduce the antioxidant density of pollen even when total pollen production increases, creating a fundamental challenge for bee nutrition in future climate scenarios.

### 4.2. Changes in Pollen Composition Affecting Its Antioxidant Levels

Environmental stress factors associated with climate change influence the production of secondary metabolites in plants, directly affecting the antioxidant content of pollen [120,121]. Plants experiencing water stress, temperature extremes, or elevated UV radiation may increase their production of protective polyphenolic compounds [115]. However, these same stress factors can also impair plant metabolism and reduce overall pollen quality [122]. The carbon-to-nitrogen ratio in plant tissues shifts under elevated atmospheric CO_2_, potentially altering the balance between structural carbohydrates and bioactive secondary metabolites [123]. This shift may reduce the concentration of nitrogen-containing antioxidant compounds while potentially increasing carbon-based polyphenols [120]. The net effect on total antioxidant capacity depends on the specific compounds affected and their relative contributions to protective activity [45]. For example, phenological shifts have been documented in Central Europe; warmer winters lead to earlier blooming of plants such as hazel [21]. Hazel is a wind-pollinated plant whose pollen, while abundant and often used by bees in late winter, generally has lower polyphenol content than insect-pollinated flowers. If climate change causes bees to rely more on such early wind-pollinated sources (due to mismatches with traditional spring flowers), their intake of antioxidants could diminish compared to a normal spring diet of willow, prunus, and brassica pollen (which are rich in phenolics) [21]. The nutritional quality and safety of pollen critically influence its protective effect on honeybee health. Pollutants such as heavy metals (e.g., lead and cadmium) and pesticide residues can contaminate pollen collected near polluted environments, compromising its antioxidant potential and exerting toxic effects. Heavy metals bioaccumulate in bee tissues, causing oxidative stress and interfering with enzymatic antioxidant defenses. Similarly, contamination with pesticides from agricultural practices presents a dual threat by directly poisoning bees and reducing the functional integrity of their diet [124]. Bees foraging in polluted air or soils demonstrate reduced survival and impaired ability to perform their ecological roles, emphasizing the need for monitoring and mitigating environmental contamination in apiary settings. Strategies for improving pollen quality include selecting foraging areas with minimal pollution, employing contamination screenings, and potentially offering uncontaminated supplemental feed to maintain nutritional adequacy and antioxidant capacity [125]. Assessment of biochemical stress markers such as heat shock proteins and antioxidant enzymes can further inform the impact of environmental contaminants on bee health and guide management interventions [126]. Soil chemistry changes associated with climate change, including altered precipitation patterns and increased weathering rates, influence plant mineral uptake and metabolism. Selenium, zinc, and other trace elements essential for antioxidant enzyme function may become less available to plants, reducing the micronutrient content of pollen [16]. These deficiencies can propagate through the food web to create antioxidant deficiencies in bee colonies [127]. Interactions between climate change and other environmental stressors may create synergistic effects on pollen composition. Air pollution, habitat fragmentation, and agricultural practices interact with climate factors to influence plant health and secondary metabolite production [128]. Understanding these complex interactions is crucial for predicting future changes in pollen antioxidant availability [14].

### 4.3. Potential Consequences for Honeybee Nutrition and Overall Vitality

Climate-mediated changes in pollen antioxidant availability may compromise honeybee nutritional status through several pathways. Reduced diversity of available pollen types limits the intake of specific antioxidant compounds that may provide complementary or synergistic protective effects [15]. Additionally, honeybees demonstrate selective foraging behavior influenced by pollen nutritional content, but may face constrained choices in degraded floral landscapes resulting from climate change [129]. Nutritional stress resulting from suboptimal antioxidant intake affects immunocompetence in honeybees facing multiple environmental stressors. DeGrandi-Hoffman et al. (2016) showed that honeybee colonies with access to diverse pollen sources exhibited greater immunological resilience to *N. ceranae* infection compared to nutritionally stressed colonies, with effects partially attributed to antioxidant intake [116]. Climate-driven nutritional deterioration may thus exacerbate pathogen pressure through compromised immune function [63]. Furthermore, climate change potentially creates multiple stressor scenarios through simultaneous alterations of temperature regimes, nutritional landscapes, and pathogen dynamics. Experimental evidence indicates that combined thermal and nutritional decline synergistically decreases honeybee survival beyond additive effects [58]. This interaction suggests that climate-mediated nutritional deterioration may be particularly detrimental when occurring concurrently with thermal stress, potentially creating population-level impacts through reduced worker longevity. Adaptation potential exists through both behavioral and physiological mechanisms. Honeybees exhibit remarkable plasticity in foraging behavior, potentially enabling them to exploit novel floral resources as plant communities reorganize under climate change [130]. Additionally, supplemental feeding with antioxidant-rich formulations may partially mitigate climate-induced nutritional deficiencies; however, the economic feasibility of widespread implementation remains uncertain [15,90,131,132,133,134].

## 5. The Role of Dietary Antioxidants in Honeybee Immunity

### 5.1. Enhancement of Immune Responses Through Dietary Antioxidants

Multiple studies showed that honeybees fed antioxidant-rich diets show elevated immune activity. As mentioned earlier, polyphenols like p-coumaric acid can upregulate genes for antimicrobial peptides. Alaux et al. (2010) found that polyphenol-supplemented diets increased the expression of abaecin and defensin, key antimicrobial peptides in honeybees, suggesting an immunostimulatory effect of dietary phenolics [29]. In a recent experiment, adding grape pomace (high in anthocyanins) to the diet not only reduced virus levels but also significantly increased the expression of Relish, a transcription factor in the NF-κB pathway that controls many immune genes [114]. This indicates that antioxidants can activate immune pathways, priming bees to respond more vigorously to infections. Likewise, bees on pollen diets (as opposed to only sugar) showed higher baseline levels of immune enzymes such as glucose oxidase (which generates antiseptic hydrogen peroxide in honey) and higher hemolymph phenol oxidase activity. Such differences are attributable to the presence of vitamins and polyphenols in natural diets that are absent in pure sugar feeds [39]. Notably, one study observed that bees fed only sucrose had the highest mortality and poor immunocompetence, whereas those consuming pollen or bee bread had lower mortality and better immune indicators despite an increase in certain oxidative stress biomarkers [39]. The same authors interpreted the elevated oxidative enzyme activities in pollen-fed bees not as pathological oxidative stress, but as an adaptive shift in redox balance due to activation of immune and detox pathways. This underscores that a nutrient-rich diet can simultaneously challenge bees (by ramping up metabolism and immune function, hence measurable ROS) but ultimately benefit them by enhancing survival and immunity, as a hormetic effect moderated by dietary antioxidants.

### 5.2. Influence on Honeybee Resistance to Diseases

Dietary antioxidants have been linked to improved resistance against a range of honeybee pathogens. For instance, *N. ceranae* infections are known to cause oxidative stress in bees and are exacerbated by poor nutrition. Polyphenols in pollen appear to help bees tolerate and suppress *Nosema* spp. Feeding experiments show that bees on polyphenol-rich diets have lower spore loads than those on polyphenol-deficient diets [114]. Similar effects were observed after additional feeding of honeybee colonies with protein additives [132,134]. This protective effect appears to be partially mediated by enhanced epithelial integrity in the midgut, the primary site of microsporidia infection, suggesting that antioxidant-mediated preservation of the peritrophic membrane barrier function occurs. Curcumin supplementation is reported to reduce *Nosema* spp. spores load and increase lifespan in infected honeybees [113]. Research shows that p-coumaric acid in the diet decreases *Nosema* spp. infection levels, potentially by upregulating immune genes that fight the parasite [42]. Extracts from native Chilean plants rich in the flavonoids rutin and myricetin significantly decreased *Nosema* spore counts and improved survival of infected bees [114]. Propolis constituents like galangin and pinocembrin (also flavonoids that bees might ingest in small amounts) were similarly associated with lower *Nosema* spp. spores loads and heightened immune response in bees [114]. Viral pathogen dynamics also demonstrate sensitivity to honeybee antioxidant status. For instance, DeGrandi-Hoffman et al. (2010) observed inverse correlations between pollen consumption and DWV titers in honeybee colonies, with effects attributed partially to antioxidant-mediated immune enhancement [116]. Resistance to parasitic mites, particularly *V. destructor*, demonstrates a link between honeybee nutritional status and antioxidant intake. Given the central role of this parasitic mite in global honeybee colony losses, these nutritional interactions warrant further investigation for potential application in integrated pest management strategies [38,135]. Field studies provide ecological validation of laboratory findings regarding antioxidant-mediated resistance to pathogens. For instance, Alaux et al. (2017) demonstrated that honeybee colonies situated in high floral diversity landscapes exhibited lower pathogen prevalence and enhanced survival compared to those in simplified agricultural landscapes, with effects partially attributed to improved nutritional status, including antioxidant intake [136]. These landscape-scale patterns highlight potential conservation strategies focusing on floral resource diversity to enhance honeybee health through dietary mechanisms.

### 5.3. Synergistic Effects of Different Antioxidants on Immune Function

Honeybees gather antioxidants from various pollens and plants, which may work together to enhance immunity. Different antioxidants affect immune defense in unique ways; for instance, flavonoids can induce detox and immune gene expression, while vitamins neutralize radicals and stabilize immune cell membranes. Research suggests that combinations of phytochemicals can have additive or synergistic benefits. An illustrative case is the interaction of quercetin and p-coumaric acid: bees fed both showed greater protection against pesticide toxicity than those fed either alone [42]. In that scenario, each phytochemical likely induced overlapping but distinct sets of detoxification genes, broadening the protective effect. Similarly, a diverse multifloral pollen diet is often superior to any single pollen type in promoting bee health, precisely because it delivers a wider array of bioactives that can cover each other’s gaps. There is also synergism between diet-derived antioxidants and the bees’ enzymatic defenses: as one increases, it bolsters the other. For example, vitamin C works synergistically with GSH and CAT, where well-nourished bees with high vitamin C levels tend to possess a more reactive glutathione system, which aids in the rapid detoxification of peroxides [21]. Diverse antioxidant compounds in the honeybee diet show synergistic effects on immune function through complementary mechanisms. Polyphenols and vitamin C exhibit well-documented interactions, with ascorbic acid restoring the antioxidant capacity of oxidized flavonoids, thereby extending their functional longevity [101]. This recycling mechanism may enhance the antioxidant capacity that supports honeybee immune function when consuming diverse pollen mixtures, potentially influencing gut microbiota composition and immunity. Jones et al. (2018) demonstrated that specific polyphenols selectively promoted beneficial microbial taxa in the honeybee gut, potentially enhancing resistance against opportunistic pathogens [117]. This microbiome-mediated mechanism represents an indirect pathway through which dietary antioxidant diversity may influence honeybee immunocompetence beyond direct effects on host physiology. The collective evidence supports nutritional strategies that emphasize pollen diversity over single-compound supplementation for optimal immune benefits. Experimental studies consistently show superior health outcomes in honeybees consuming polyfloral compared to monofloral pollen sources, with effects partially attributed to broader antioxidant profiles [6]. These findings have direct implications for both conservation strategies focusing on floral diversity and the potential development of antioxidant-enriched supplemental feeds for managed honeybee colonies, as part of good beekeeping practices and biosecurity measures.

## 6. Antioxidants and Honeybee Resilience to Environmental Stressors

Beyond their classical nutritional constituents, pollen polyphenols constitute key antioxidant agents in the honeybee diet. These compounds help mitigate oxidative damage induced by environmental stressors, thereby enhancing bee longevity and colony sustainability. Polyphenol-rich diets have been shown experimentally to improve food consumption and detoxification capacity in worker bees exposed to pesticides, reflecting their role in modulating antioxidant defense systems and promoting survival under toxic insults [43]. Dietary supplementation with phenolic acids and flavonoids has been linked to the upregulation of detoxifying enzymes, including cytochrome P450s and antioxidant enzymes, indicating that these bioactive compounds prime the bees’ physiological defenses. Such nutritional strategies can counteract immune suppression and oxidative damage caused by environmental toxins, supporting the use of polyphenol-enriched pollen in apicultural nutrition [137]. However, pollen’s composition is influenced by species, climatic and soil factors, thus affecting polyphenol bioavailability, and necessitating further research to optimize formulations for maximal health benefits [138].

### 6.1. Protection Against Pesticide-Induced Oxidative Stress

Dietary antioxidants provide significant protection against pesticide toxicity in honeybees through multiple mechanisms. Neonicotinoid insecticides represent the most extensively studied pesticide class concerning honeybee oxidative stress [23]. These compounds appear to disrupt mitochondrial electron transport chains, increasing ROS production while simultaneously depleting cellular glutathione levels [139]. Bees with enhanced antioxidant status through dietary supplementation show improved survival rates and reduced behavioral impairments when exposed to sublethal neonicotinoid concentrations [140]. As it was mentioned earlier, pesticides are significant stressors that contribute to declines in honeybee populations, causing neurotoxic and oxidative effects. Pyrethroids, neonicotinoids, and fungicides induce oxidative stress by generating ROS and impairing mitochondrial function, overwhelming endogenous antioxidant defenses. Honeybees deploy cytochrome P450 monooxygenases, glutathione-S-transferases, and other phase I/II detoxification enzymes to metabolize and eliminate such xenobiotics [141]. Notably, polyphenols, such as those found in bergamot polyphenolic fractions, have demonstrated protective effects by reducing mortality in bees exposed to deltamethrin, a pyrethroid pesticide. Such polyphenolic antioxidants mitigate oxidative stress and compensate for energy deficits caused by pesticide-induced metabolic perturbations [141]. Furthermore, nutritional supplementation with polyphenol-rich pollen has been linked to improved detoxification gene expression and survival in pesticide-exposed bees, evidencing a potential nutritional approach to mitigate chemical stress [43]. The combination of understanding detox pathways and integrating antioxidant dietary components forms a critical strategy for protecting the bees from ongoing chemical challenges. Organophosphate and carbamate insecticides create oxidative stress through different mechanisms, primarily by inhibiting acetylcholinesterase and disrupting normal neurotransmission [142,143]. The resulting hyperexcitation increases metabolic demands and ROS production in neural tissues. Antioxidant protection appears particularly important for maintaining cognitive functions essential for navigation and communication under these conditions. Herbicide exposure, while generally less acutely toxic than insecticides, may create chronic oxidative stress through disruption of plant communities and reduction in dietary antioxidant availability. Glyphosate and other widely used herbicides can alter the polyphenolic content of plants, indirectly affecting bee antioxidant nutrition. These indirect effects may be more significant for long-term bee health than direct herbicide toxicity [144]. Fungicide interactions with bee antioxidant systems appear particularly complex, as many fungicides are designed to disrupt cellular respiration in target organisms. While bees possess different cellular machinery than fungi, some fungicides may affect bee mitochondrial function and antioxidant enzyme activity [64]. Synergistic interactions between fungicides and other pesticides may create unpredictable effects on bee oxidative stress levels [145]. Mechanistically, pollen polyphenols enhance detoxification capacity by upregulating metabolic enzymes. P-coumaric acid significantly increases the expression and activity of cytochrome P450 monooxygenases, particularly CYP9Q1, CYP9Q2, and CYP9Q3, which metabolize a wide range of pesticides, including neonicotinoids and organophosphates [8]. Schmehl et al. (2014) showed that honeybee colonies with access to diverse pollen sources experienced reduced mortality following field-realistic pesticide exposure compared to nutritionally restricted colonies [146]. This practical demonstration of antioxidant-mediated resilience highlights potential management strategies emphasizing nutritional interventions to mitigate pesticide impacts in agricultural settings.

### 6.2. Role in Mitigating Stress from Climate Change and Habitat Loss

With anticipated increases in winter temperature variability due to climate change, antioxidant nutrition could be a crucial factor affecting overwintering success and spring honeybee population strengthening. One indirect evidence is that well-nourished “winter bees” (long-lived bees produced in the autumn) have exceptionally high vitellogenin and antioxidant enzyme levels, which help them survive months of cold [20]; winter bees come from colonies that had good late-season pollen (like goldenrod and aster) rich in antioxidants, supporting the idea that diet contributes to climate resilience. Research demonstrates that pollen polyphenols offer broad protective effects against various environmental stressors that threaten honeybee health and survival. These bioactive compounds exhibit notable antioxidant, anti-inflammatory, anti-mutagenic, anti-allergic, antimicrobial, and antitumor properties, with most of these protective activities linked to their phenolic content and radical scavenging capacity [71,147,148]. The antioxidant potential of pollen extracts has been shown to rival that of pure antioxidant compounds such as quercetin and ascorbate, demonstrating robust capacity for neutralizing harmful free radicals through multiple mechanisms, including iron reduction and radical scavenging [149]. UV radiation represents a significant environmental stressor that can damage bee reproductive systems, but pollen polyphenols provide crucial protection during these conditions. Studies show that flavonoids, particularly flavonols and anthocyanins, promote healthy pollen tube growth by lowering reactive oxygen species levels and mitigating their adverse effects on cellular function [107,108]. Heat stress similarly increases ROS levels in reproductive tissues, but flavonols prevent these reactive species from reaching damaging concentrations, thereby maintaining fertility and reproductive success even under elevated temperatures [108]. The protective effectiveness of pollen polyphenols varies significantly with environmental conditions and collection timing, highlighting the importance of optimal management practices. Environmental factors such as soil conditions and plant physiology impact the antioxidant capacity of bee pollen, while collection during UV-intense periods from early to late summer yields pollen with higher antioxidant activity [68,70]. Proper storage methods are also critical for maintaining protective compounds, with freezing at −20 °C proving most effective for preserving vitamin C, polyphenols, and antioxidant properties compared to drying methods that can cause significant degradation [150]. Habitat fragmentation and resulting nutritional stress interact with antioxidant physiology to influence honeybee resilience. Honeybee colonies in simplified agricultural landscapes show reduced antioxidant enzyme activity and increased oxidative damage markers compared to those in diverse habitats, impacting disease resistance and longevity [136]. These findings highlight the importance of landscape-scale conservation efforts that preserve floral diversity to support honeybee nutritional needs, including antioxidant intake. Beyond direct antioxidant effects, pollen polyphenols provide systemic health benefits that support overall stress resistance in honeybees. These compounds can modulate cardiovascular-like systems by improving endothelial function, reducing inflammatory cytokine synthesis, and enhancing nitric oxide production, which collectively support physiological resilience under environmental pressure [151]. The broad spectrum of biological activities exhibited by bee pollen polyphenols positions them as natural protective agents that can help honeybee colonies cope with the mounting environmental challenges they face in modern ecosystems. To counteract these environmental deficits, nutritional interventions may mitigate climate and habitat-related stressors through antioxidant supplementation. Fleming et al. (2015) demonstrated that supplemental feeding with polyphenol-enriched formulations increased heat tolerance and reduced oxidative damage in honeybees, suggesting practical applications for beekeeping in changing climates [152]. However, economic constraints and limited understanding of appropriate supplementation protocols currently restrict widespread implementation of these approaches.

### 6.3. Effects on Adult Bees’ Longevity and Colony Survival

The ultimate metrics of resilience are how long individual bees live and whether the colony as a whole survives and thrives. Antioxidant intake has a pronounced impact on both. Worker bees on pollen diets live significantly longer than those on protein-poor diets and numerous studies confirm that longevity is linked to diet quality and antioxidant levels [21,39]. For instance, bees fed polyphenol-enriched diets (through natural pollen or spiked supplements) had an extended lifespan even under stress exposure [42]. One mechanism is the preservation of physiological function with age: oxidative damage is a key driver of aging, so antioxidants slow the accumulation of such damage. Additionally, some antioxidants like vitellogenin (which has antioxidant properties and is tied to pollen intake) act as life-extending factors in bees [6,153]. On a colony level, good nutrition with ample antioxidants translates to higher colony population and survival rates. Colonies situated in diverse floral landscapes (e.g., wildflower meadows) typically show higher brood viability, greater adult population heading into winter, and better overwintering success than those in poor landscapes [111]. Colonies with access to pollen high in antioxidants had lower off-season mortality and were more robust against parasites compared to those fed mostly monoculture pollen or syrup. Adding antioxidant supplements to colonies has improved markers of colony health, for example, increased brood rearing and lower *Nosema* spp. infection rates, and higher winter survival percentages [6,39,116,154]. Winter bees, whose extended lifespan is crucial for the success of colony overwintering, demonstrate particular sensitivity to antioxidant status. Colonies fed nutrient-rich autumn pollen showed a winter survival rate above 90%, significantly larger brood area, and in vitro worker longevity extended to about 23 days, contrasting sharply with honeybee colonies fed syrup alone. These results suggest that autumn pollen quality, likely enriched with micronutrients such as vitamin E and other antioxidants, influences the physiological capacity of winter bees and predicts spring colony strength [155]. Colony-level survival studies provide ecological validation of experimental findings regarding antioxidant-mediated resilience. Smart et al. (2016) demonstrated that apiaries situated in landscapes with greater floral diversity experienced reduced annual mortality compared to those in poor agricultural environments, with effects partially attributed to nutritional factors, including antioxidant intake [156]. These landscape-scale patterns highlight potential conservation and management strategies focusing on nutritional resources to enhance honeybee colony survival. The relationship between worker longevity and colony success creates a critical feedback loop for colony survival. When environmental stressors trigger precocious foraging behavior, shortened worker lifespans can prevent colonies from maintaining adequate populations to support larval development, ultimately leading to colony death [157]. Adequate pollen access prevents this cascade by supporting normal worker development, including enhanced hypopharyngeal gland function and improved ovarian development, which directly impacts the colony’s ability to rear brood successfully [158]. Table 3 highlights the relationship between pollen-derived polyphenols, environmental stressors, and related antioxidants linked to health outcomes.

## 7. Knowledge Gaps and Future Research Directions

Despite progress in understanding dietary antioxidants’ role in honeybee health, significant gaps remain. One key issue is the lack of clear dose–response relationships between antioxidant intake and physiological outcomes. While compounds like flavonoids and phenolic acids show protective effects, optimal intake levels and toxicity thresholds are not well defined. Additionally, the absence of standardized methods and metrics hinders conclusive findings across studies. Investigations involving controlled dietary manipulations, graduated concentration ranges, and multiple biomarkers would enable a more mechanistic understanding of how varying antioxidant levels influence immune function, detoxification, longevity, and stress resistance. Such data are essential for formulating evidence-based nutritional guidelines, particularly for beekeepers managing colonies under stress from agricultural intensification or climate variability [6] and veterinarians as crucial stakeholders in animal health under the One Health approach [159]. In addition to intake uncertainties, the molecular mechanisms underlying antioxidant absorption, transport, and utilization in bee physiology remain incompletely characterized [160]. Advanced metabolomics and transcriptomics approaches could provide insights into how different antioxidant compounds are processed and distributed within bee tissues [161]. Few studies have investigated the correlation between different antioxidant doses and physiological endpoints under laboratory-controlled conditions. Current research often focuses on the presence or absence of antioxidants rather than quantifying optimal levels for different physiological states and environmental conditions [6]. Such quantitative clarity would directly inform practical supplementation protocols for managed colonies [86]. The temporal dynamics of antioxidant protection present another area requiring focused research attention [162]. Questions remain regarding how long antioxidant protection persists after consumption, whether there are critical windows when antioxidant supplementation provides maximum benefits, and how antioxidant requirements vary across bee life stages and seasonal cycles [15]. Synergistic and antagonistic interactions among different antioxidant compounds need comprehensive characterization [44]. While some beneficial interactions have been identified, the full complexity of antioxidant interactions in bee nutrition remains unexplored [45]. Understanding these interactions could inform strategies for optimizing antioxidant mixtures rather than focusing on individual compounds. Environmental factors affecting antioxidant bioavailability and efficacy require systematic study [121]. Temperature, humidity, UV exposure, and other environmental variables may influence how effectively bees can utilize dietary antioxidants [120]. These factors become particularly important as climate change alters environmental conditions in bee habitats [115]. The economic implications of antioxidant nutrition for commercial beekeeping operations need quantitative assessment [128]. While enhanced health and productivity appear likely to provide economic benefits, the costs and benefits of different antioxidant intervention strategies require careful analysis to guide commercial adoption [14]. Standardization of methods for measuring antioxidant status in bees and bee products is also urgently needed. Current methods vary substantially among research groups, making it difficult to synthesize findings and establish reference values for different conditions [127,137]. The development of validated biomarker panels, for both field and laboratory use, would significantly enhance the coherence and reproducibility of research findings. Understanding age- and caste-specific nutritional demands could inform strategic feed supplementation during critical phases of colony development, including early brood rearing, drone production, or queen rearing [86].

Another important and unresolved issue is the nature of interactions among antioxidant compounds. While some combinations of polyphenols and flavonoids appear to produce synergistic effects that enhance antioxidant capacity beyond that of individual components, other studies suggest antagonism, where certain compounds interfere with absorption or efficacy when co-administered [29,163]. Such conflicting results underscore the need for systematic evaluation of compound interactions across different doses and stress contexts. This includes assessing additive versus synergistic outcomes and determining whether particular combinations may inadvertently reduce protective benefits or cause redox imbalance. A critical shortcoming of the current literature is its reliance on simplified experimental models that do not capture the multifactorial nature of stress in the real-world environments of honeybee colonies. Most studies isolate a single stressor, such as pesticide exposure or poor nutrition, while in nature, colonies often face multiple simultaneous challenges, including pathogens, chemical contaminants, temperature fluctuations, and forage scarcity. More field studies that incorporate these multifactorial stressors and measure antioxidant dynamics across the season are needed to better understand how nutritional strategies interact with broader ecological pressures [133]. Such research would provide more relevant insights into the resilience of honeybee colonies in commercial and conservation settings. Compounding these knowledge gaps is the limited understanding of the bioavailability and metabolism of ingested antioxidants in honeybees. While certain phenolic compounds, such as p-coumaric acid, are known to be absorbed and utilized by honeybees [8], most dietary antioxidants have not been pharmacokinetically characterized in this species. Critical questions remain about how these compounds are processed post-ingestion, how they are distributed across tissues, whether they are metabolically activated or degraded, and what biological roles their metabolites might serve. The honeybee gut microbiota likely plays an underappreciated role in modulating the efficacy of antioxidants. Several core microbial species possess enzymatic capacities to degrade or transform dietary phytochemicals, potentially altering their biological activity or facilitating absorption [164]. The extent to which these microbial transformations enhance or diminish antioxidant effects in vivo remains unknown. Studies using germ-free bees or controlled microbial reconstitution models could illuminate the symbiotic role of gut microbes in host antioxidant defense, paving the way for microbiome-informed dietary interventions. Methodological inconsistency is a pervasive issue in this field. Current studies rely on a diverse array of assays to evaluate oxidative status and antioxidant function, including enzymatic activity measurements (e.g., SOD and CAT), assessments of oxidative damage (e.g., lipid peroxidation markers), and direct quantification of antioxidant compounds in tissues. This variability hinders cross-study comparisons and slows the meta-analytical synthesis process [165]. The development of standardized biomarker panels and validated assays for field and laboratory use would significantly enhance the coherence and reproducibility of research findings. Technological challenges also hinder the translation of antioxidant research into practical beekeeping solutions. Antioxidant supplements are currently limited by issues such as compound instability, poor palatability, and uncertain bioavailability. Beyond biological and technical issues, socioeconomic and practical barriers must be addressed to facilitate the adoption of antioxidant supplementation in commercial beekeeping. Despite promising laboratory evidence, few studies have evaluated the cost-effectiveness of these interventions at scale. Integration with existing management routines, such as feeding schedules, anti-varroa treatments, and queen replacement protocols, requires logistical compatibility and minimal labor burden. Collaborative research with beekeepers and other stakeholders in apiculture, as well as involvement from the early stages of supplement development, can help ensure that the resulting products meet the needs and constraints of consumers [86]. Ultimately, aligning research priorities with industry perspectives is key to driving meaningful application. In summary, advancing antioxidant-based approaches to honeybee health and welfare will require a more integrated, mechanistic, and translational research agenda. Addressing dose-dependent effects, metabolic pathways, compound interactions, and field-relevant stress dynamics, while simultaneously resolving practical and economic concerns, will position antioxidant nutrition as a viable tool in the broader strategy to enhance pollinator resilience in a changing world (Table 4).

When evaluating the use of pollen supplementation for honeybee colonies, it is essential to address several practical aspects. The optimal delivery method (microencapsulation, protein patties, or liquid suspensions) remains unresolved, as each technique offers distinct advantages for nutrient stability and acceptability. It is advisable to limit pollen harvesting to preserve natural food stores and enhance honeybee colony resilience. Additionally, while supplemental feeding during the summer may help address nutritional resource shortages, further research is needed to evaluate its potential effects on the quality of honey and royal jelly.

## 8. Conclusions

Pollen-derived polyphenols are fundamental to honeybee health, providing essential antioxidant defenses that bolster immunity, detoxification pathways, and overall resilience against a suite of environmental stressors. The availability of these crucial compounds, however, is increasingly threatened by anthropogenic and environmental pressures, including climate change and habitat degradation, which disrupt floral resources. This review synthesizes the evidence suggesting that a diet rich in diverse polyphenols is not merely beneficial but may be critical for colony survival in challenging environments. Therefore, it is crucial to integrate antioxidant-focused nutritional strategies into conservation frameworks and practical beekeeping management. Future efforts must be directed toward translating this biochemical understanding into tangible applications, such as the development of targeted, evidence-based feed supplements and the promotion of floral diversity, to support the long-term health and sustainability of these vital pollinators.

## Figures and Tables

**Table 1:** Summary of antioxidant endpoints connected with pollen diets and honeybee health outcomes.

Direct ROS Assay	Enzyme Activity (SOD/CAT/etc.)	Oxidative Damage Markers	Gene/Protein Expression	Link to Polyphenol/Pollen	Observed Health Outcome	Ref-(s)
no	GST (activity), P450, vitellogenin (expression)	no	yes	yes	not specified	[37]
no	SOD, CAT, GST, GPx	no	no	yes (pollen blends compared)	survival, pesticide clearance	[7]
no	no	no	yes (by pollen type)	yes	none—enzymatic levels only	[21]
TBARS, protein carbonyls	CAT	TBARS, carbonyls	yes (transcriptomics)	yes (fractionated pollen)	survival, overwintering	[38]
not specified	antioxidant capacity	not specified	lysozyme (protein)	no (pollen present; not polyphenol-focused)	survival	[39]
no	not detailed	no	some detox gene upregulation	limited	survival, longevity, digestion, immunity	[40,41,42,43]

**Table 2:** A summary of the effects and consequences of oxidative stress in honeybees.

Category	Effect	Mechanism/Description	Ref-(s)
molecular level	↑ ROS	overproduction of O_2_^−^, H_2_O_2_, OH· during mitochondrial respiration	[3,15]
	lipid peroxidation	oxidative degradation of membrane lipids → cell damage	[60]
	protein oxidation	oxidative modification of amino acids; impaired enzyme function	[24]
	DNA damage	strand breaks, base modifications may impair gene expression	[61,62]
cellular response	antioxidant enzyme activation	upregulation of SOD, CAT, GPx to detoxify ROS	[17]
	depletion of non-enzymatic antioxidants	GSH, vitamin C, vitamin E are used to neutralize ROS	[24]
neural function	cognitive impairment	ROS damage to neurons → navigation and learning deficits	[51]
	neurodegeneration	structural brain damage in foragers exposed to pesticides	[60]
immune function	suppressed immunity	reduced hemocyte function and antimicrobial peptides	[55]
	↑ susceptibility to pathogens	*Nosema* spp. and viruses proliferate under oxidative stress	[29,57]
	impaired immune gene expression	disrupted expression of immune-related genes (e.g., defensins)	[57]
reproduction	queen fertility decline	exposure to thiamethoxam is linked to oxidative stress in the ovaries	[31]
	queen replacement	behavioral response to declining queen performance	[59]
metabolism and longevity	accelerated aging	especially in winter bees; shorter lifespan due to ROS accumulation	[54]
	↑ metabolic load in foragers	foraging increases ROS via mitochondrial respiration	[15]
colony-level effects	disrupted division of labor	leads to precocious foraging; it weakens the colony’s age structure	[58]
	reduced brood production	nutritional and physiological stress impairs brood care	[57]
	lower honey yield	impaired foraging and navigation → reduced nectar collection	[33]
	colony mortality	combined effects of oxidative stress, pathogens, and poor nutrition	[14,57]
environmental triggers	pesticides (e.g., neonicotinoids)	imidacloprid, thiamethoxam increase ROS, suppress antioxidants	[24,60]
	pathogens (DWV, ABPV, *Nosema* spp.)	induce host ROS and weaken antioxidant systems	[27,29]
	nutritional stress	lack of diverse pollen leads to antioxidant deficiency	[33,63]
	high temperature/climate change	enhances ROS production; disrupts bee physiology	[14]
	RF-EMFs (radiofrequency electromagnetic fields)	transport and hive movement increase oxidative load	[36,37]
		induce oxidative biomarkers in bee tissues	[35]
protective role of diet	pollen-derived polyphenols	antioxidant activity reduces ROS, supports detoxification	[64,65]
	vitamin C and E supplementation	enhance resilience against pesticide-induced oxidative stress	[24]
	floral diversity	pollen sources improve antioxidant enzyme expression	[33]

Arrows show the increase, or sequential steps of the reaction/process.

**Table 3:** Summary of polyphenol pollen source links with tested environmental stressors and antioxidants/health endpoints.

Polyphenol Source/Characterization	Pollen as an Experimental Variable	Stressor(s) Tested	Antioxidant/Redox Endpoints	Health/Outcome Endpoints	Link(s)	Ref-(s)
+ pollen quantified for p-coumaric acid	+ two distinct pollen blends; measured phenolics	pesticides (azoxystrobin, sulfoxaflor)	GST, P450 gene expression, vitellogenin (expression only); no direct redox damage assays	none directly measured	(−) direct redox damage assays	[37]
+ monofloral pollens (rapeseed, phacelia, etc.); phenolic/flavonoid content noted	+ 6 monofloral pollens vs. sugar-only	nutritional stress (monodiet)	SOD, CAT, GST, GPx (activity) in hemolymph/fat body	survival, pesticide clearance	+ polyphenol composition-dependent resilience	[7]
+ pollen fractionated (apolar vs. polar)	+ pollen vs. sugar	*V. destructor*, DWV	None beyond enzymatic shifts	none beyond enzymatic levels	(weak) no classic stressor link, but polyphenol-diet effect observed	[21]
not quantified; pollen and bee bread diets	+ rapeseed/willow pollen and bee bread vs. controls	none applied directly	TBARS, protein carbonyls, CAT activity, lysozyme	survival (lab/field), overwintering, gene expression changes	(weak) survival benefit lost with apolar fraction removal; no redox markers	[38]
+ detailed for 3 pollen types, polyphenol content compared	+ maize, lotus, sunflower pollen	none applied directly	no mechanistic linkage to polyphenols; more diet-induced redox status	digestion, immunity, gut microbiota	(−) polyphenol-focused	[39]
(−) pure compounds (e.g., quercetin, p-coumaric acid) at pollen-level doses	(−) compounds in artificial diets, not pollen	pesticides (imidacloprid, propiconazole, thiacloprid, boscalid)	detox gene upregulation (CYP, GST); no ROS/damage metrics	survival, longevity, food intake, performance	limited: antioxidant effect presumed, no in vivo polyphenol pollen link	[40,41,42,43]

Note: + means yes; (−) means no; (weak) means weak connection.

**Table 4:** Summary of knowledge gaps and future research directions in honeybee antioxidant nutrition.

Research Area	Knowledge Gap/Challenge	Future Direction	Ref-(s)
dose–response relationships	undefined optimal intake, efficacy thresholds, and potential toxicity of antioxidants	controlled dose trials using multiple biomarkers and stressors	[6,134,166]
developmental and caste-specific needs	limited data on stage- and caste-specific antioxidant demands (larvae, queens, drones)	formulation of life-stage-targeted antioxidant diets	[17,167]
compound interactions	unclear whether combinations of antioxidants are synergistic or antagonistic	systematic evaluation of common polyphenol/flavonoid interactions	[15,163]
real-world multifactorial stress models	laboratory studies often isolate a single stressor; field-relevant complexity is missing	seasonal field studies simulating pesticide–pathogen–nutrition–climate combinations	[90,133,168]
bioavailability and metabolism	poor understanding of absorption, transformation, and excretion of dietary antioxidants	use of isotope-labeled compounds and LC-MS/MS profiling	[4,169]
role of microbiota	the gut microbiota interaction with antioxidant metabolism is not well characterized	germ-free bee studies; microbiome-targeted diet design	[164]
standardization of methods	inconsistent biomarkers and oxidative stress assays limit comparability	development of validated standardized testing protocols and field kits	[165]
delivery technologies	antioxidants may degrade, taste bad, or be poorly absorbed	nanoencapsulation, slow-release formulations, pH-stable carriers	[170,171,172]
socioeconomic and practical adoption	lack of cost-effectiveness data and field-scale validation	participatory research with beekeepers; practical feeding protocols	[86]
precision nutrition and genetics	unknown variability in antioxidant metabolism across colonies or strains	breeding programs selecting enhanced redox resistance traits	[2,17]

## Data Availability

No new data were created or analyzed in this study. Data sharing is not applicable to this article.
